# Cacna2d2 inhibits axonal regeneration following surgical decompression in a rat model of cervical spondylotic myelopathy

**DOI:** 10.1186/s12868-022-00727-7

**Published:** 2022-07-01

**Authors:** Peisheng Liu, Xiaofeng Li, Jing Liu, Hengjia Zhang, Zhitao You, Jianfeng Zhang

**Affiliations:** 1 Department of Spinal Surgery, Yantaishan Hospital, No. 10087, Keji Avenue, Laishan District, 264000 Yantai, China; 2 Basic Department, Yantai Vocational College, 264000 Yantai, China

**Keywords:** Gabapentin, 5HT, GAP43, Iba-1, Caspase-3

## Abstract

**Background:**

Cervical spondylotic myelopathy (CSM) is a clinically symptomatic condition due to spinal cord compression, leading to spinal cord dysfunction. Surgical decompression is the main treatment of CSM, but the mechanisms of axonal regeneration after surgical decompression are still fragmentary.

**Methods:**

In a rat model of CSM, the cacna2d2 (α2δ2) expression levels in anterior horn of spinal cord were observed following compression and decompression by western blot and immunofluorescence. The expression levels of 5 hydroxytryptamine (5HT) and GAP43 were also analyzed by immunofluorescence. Furthermore, gabapentin intervention was performed for 4 weeks after decompression to analyze the changes of behaviors and anterior horn of spinal cords.

**Results:**

Following decompression, the expression levels of α2δ2 in the anterior horn of spinal cord were decreased, but the expression levels of 5HT andGAP43 were increased. Compared with the vehicle treated rats, gabapentin treatment for 4 weeks ameliorated the behaviors of rats and improved the damaged anterior horn of spinal cord. Besides, inhibition of α2δ2 through gabapentin intervention enhanced the axonal regeneration in the anterior horn of damaged spinal cord.

**Conclusions:**

Inhibition of α2δ2 could enhance axonal recovery in anterior horn of damaged spinal cord induced by CSM after surgical decompression, providing a potential method for promoting axon regeneration following surgery.

## Backgrounds

Cervical spondylotic myelopathy (CSM) commonly occurs in people aged 40–60, accounting for about 10−15% of cervical spondylosis [1, 2]. The symptoms of CSM are changeable and the etiologies are complex, resulting in difficult to explain its natural course [2]. Although the development of CSM is relatively slow, it could lead to spinal cord dysfunction and seriously affect the quality of patients’ life, even lead to disability [2, 3]. At present, surgical decompression is the main treatment of CSM, which aims to remove compression of the spinal cord, so as to effectively restore the physiological curvature of the cervical spine and the corresponding spinal canal capacity and shape. Although surgical decompression could improve spinal cord function and prevent development or deterioration of CSM, there are many postoperative complications, such as cerebrospinal fluid leakage, nerve root paralysis and changes of bone graft internal fixation [4–6]. Therefore, it is of great clinical significance to develop neuroprotective strategies as a supplementary method for CSM decompression.

Many studies have shown that CSM affects the lateral funiculi containing the lateral corticospinal tracts, resulting in axonal loss [7–9]. Lesioned axons could not regenerate in the central nervous system of adult mammals, which limits the recovery after injury. However, activation of transcriptional programs after peripheral nerve injury (PNL) allows dorsal root ganglion (DRG) neurons to produce a strong regenerative response to the second injury of peripheral or central axons [10, 11]. Dhillon et al. found that axonal plasticity was found during the recovery of injured spinal cord following surgical decompression in a rat model of CSM [9]. And, surgical decompression partially attenuated amyloid precursor protein (APP) expression and increased GAP43 expression [9]. However, the mechanisms of axonal regeneration after surgical decompression in the CSM are still fragmentary.

In mammals, encoding Alpha2delta (α2δ) subunits have been identified, including Cacna2d1 (α2δ1), Cacna2d2 (α2δ2), Cacna2d3 (α2δ3) and Cacna2d4 (α2δ4) [10, 12].α2δ (1–3) genes are differentially expressed in neurons, and α2δ4 is mainly expressed in non-neuronal cells [12]. In trafficking of Cav2 channels, α2δ subunits are required and high expression of α2δ2 increases the Cav2 channel density in the presynaptic active region [13–15]. Gabapentin or pregabalin is a potent neuropathic analgesic, and its targets are α2δ1 and α2δ2 [15, 16]. In adult mice after spinal cord injury, α2δ2 pharmacological blockade through pregabalin administration enhanced axonal regeneration [10]. However, the roles of α2δ2 in axonal regeneration in CSM following surgical decompression have not reported.

In this study, we put forward a hypothesis that α2δ2 plays a negative regulator of axonal growth and regeneration following surgical decompression in CSM, and inhibition α2δ2 after surgical decompression might enhance the axonal regeneration.

## Results

### Changes of α2δ2 expression in damaged spinal cords following surgical decompression

**Fig. 1:**
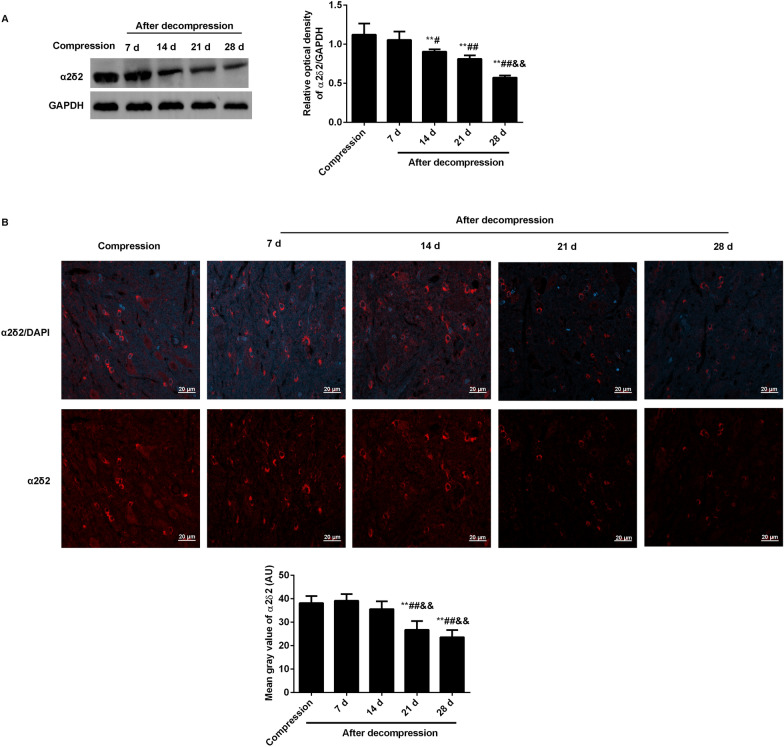
Expression levels of α2δ2 were observed in damaged spinal cord caused by a rat model of CSM. **A** The levels of α2δ2 in damaged spinal cords were analyzed at compression 5 weeks, decompression 7 days, 14 days, 21 days, and 28 d by western blot; **B** The expression of α2δ2 in anterior horn of damaged spinal cord was observed by immunofluorescence (Scale bar = 20 μm). The quantitative analysis of results was analyzed by Image J software. Compared with compression, ^**^P < 0.01; compared with decompression 7 d; ^#^P < 0.05, ^##^P < 0.01; compared with decompression 14 d, ^&&^P < 0.01

The expression levels of α2δ2 in damaged spinal cords at compression 5 weeks, decompression 7 d, 14 d, 21 d and 28 d were separately observed by western blot (Fig. 1A). Compared with the compression rats, the levels of α2δ2 were significantly reduced at 14 days after surgical decompression (Fig. 1A). In addition, the expression levels of α2δ2 in anterior horn of damaged spinal cords were observed by immunofluorescence (Fig. 1B). Consistent with the results of western blot, the tendency of α2δ2 expression was declined with the decompression time. At 21 days after decompression, the mean gray values of α2δ2 began to significantly decrease.

### Changes of 5HT and GAP43 expression in anterior horn of damaged spinal cords following surgical decompression

**Fig. 2:**
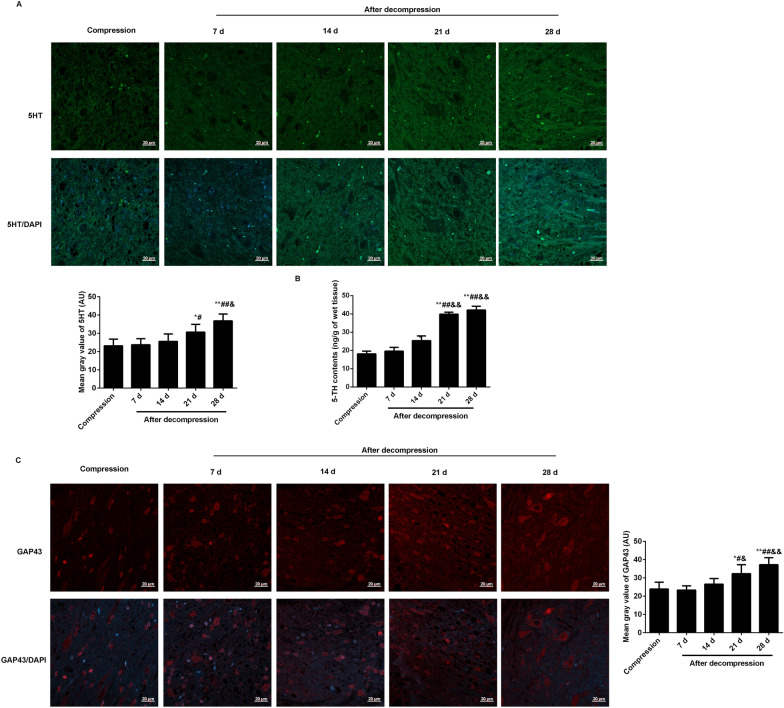
Expression levels of 5HT and GAP43 in the anterior horn of damaged spinal cord was analyzed at compression 5 weeks, decompression 7, 14, 21d, 28 d after surgical decompression in a rat model of CSM. The expression of 5HT was observed by immunofluorescence **A** and ELISA (**B**). The expression of GAP43 was observed by immunofluorescence (**C**). The mean gray value of 5HT and GAP43 was analyzed by Image J software. Compared with compression, ^*^P < 0.05, ^**^P < 0.01; compared with decompression 7 d; ^#^P < 0.05, ^##^P < 0.01; compared with decompression 14 d, ^&^P < 0.05, ^&&^P < 0.01

In the anterior horn of damaged spinal cords, 5HT and GAP43 expression levels were observed at compression 5 weeks, decompression 7 d, 14 d, 21 d, 28 d through immunofluorescence (Fig. 2). The expression levels of 5HT and GAP43 were continuously increased following surgical decompression. Contrasted to the compression rats, the levels of 5HT (Fig. 2A, B) and GAP43 (Fig. 2C) were significantly increased began to decompression 21 days.

### Gabapentin administration improved behaviors evaluation following surgical decompression

**Fig. 3:**
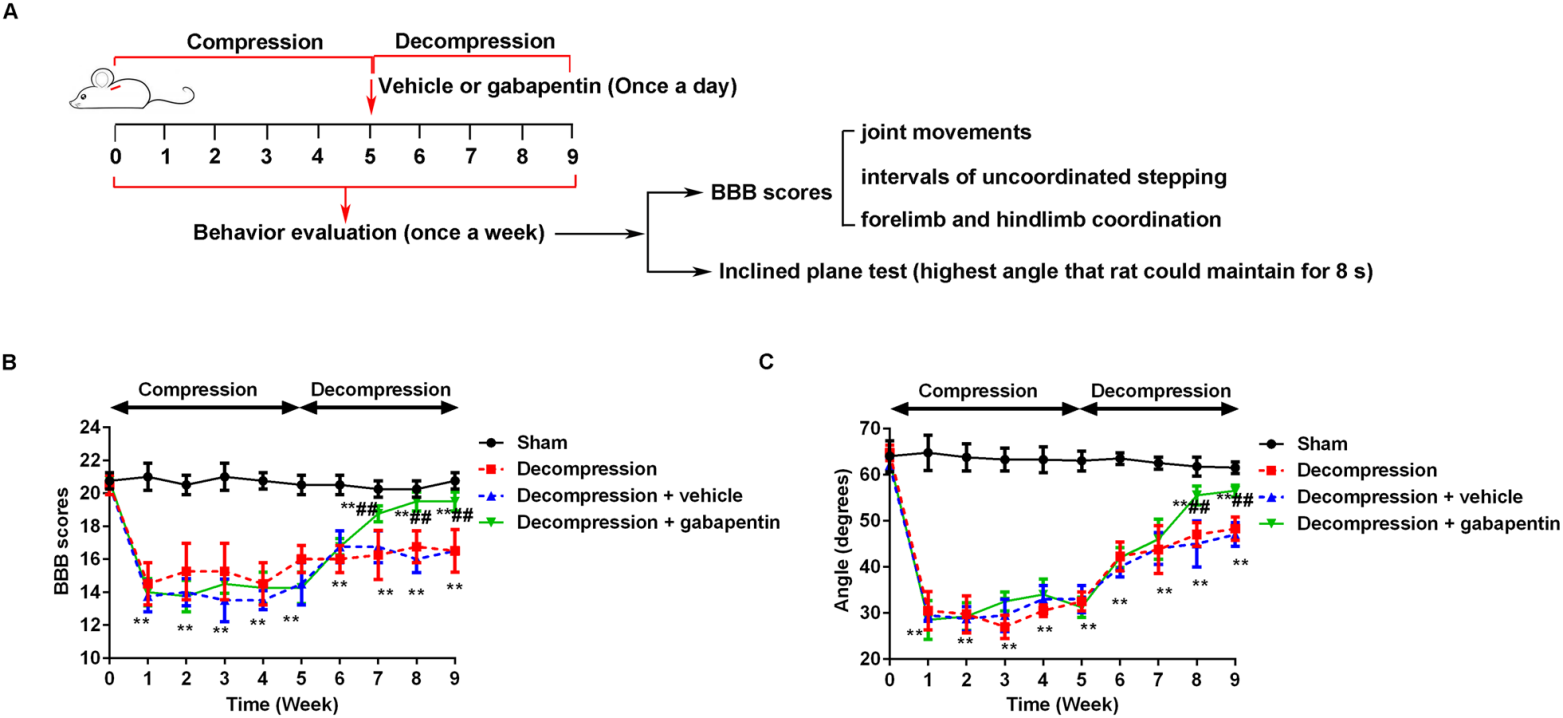
Gabapentin administration ameliorated the behaviors of rats following surgical decompression. **A** A schematic of behavior evaluation in this study. **B** The BBB scores were measured very week. **B** The maintained highest angle was measured very week. Compared with the sham group, ^**^P < 0.01; compared with the decompression + vehicle group, ^##^P < 0.01

As shown in Fig. 3A, behaviors evaluation of rats was performed in the progress of decompression and decompression once a week by BBB sores and inclined plane test. Compared with the sham group, the BBB scores (Fig. 3B) and the maintained highest angle (Fig. 3C) were significantly decreased after compression. After compression 5 weeks, rats underwent surgical decompression and vehicle or gabapentin treatment. With the time of decompression, the BBB scores and the angle were gradually increased. By contrast with the vehicle condition, gabapentin treatment promoted the behaviors recovery through increasing the BBB scores and the maintained highest angle.

### Gabapentin administration improved recovery of injured spinal cord following surgical decompression in CSM rats

**Fig. 4:**
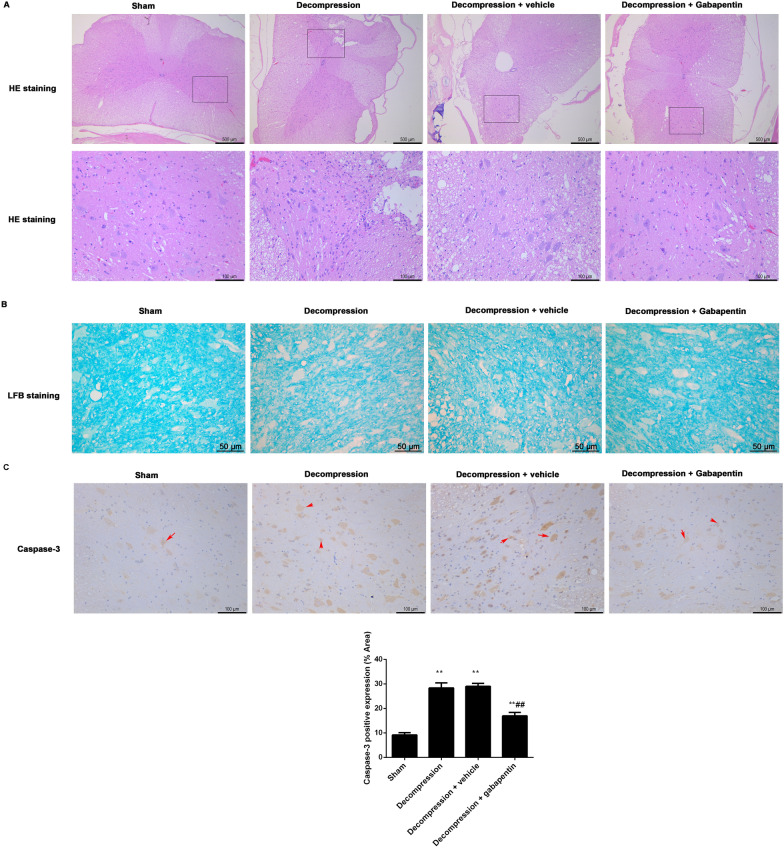
Gabapentin administration promoted recovery of injured spinal cord following decompression in CSM rats. **A** The pathological changes of anterior horn in spinal cord were observed by HE (scale = 500 μm, 100 μm). **B** The changes of axons in the anterior horn of spinal cord were measured by LFB staining (scale = 50 μm). **C** The levels of caspase-3 (red arrows) in the anterior horn of spinal cord were analyzed by immunohistochemistry (scale = 100 μm). The positive expression of caspase-3 was analyzed by Image J software. Compared with the sham group, ^**^P < 0.01; compared with the decompression + vehicle group, ^##^P < 0.01

After administration of gabapentin or vehicle for 4 weeks following decompression, the rats were sacrificed to obtain spinal cord. The pathological differences of spinal cord among groups were analyzed through HE staining (Fig. 4A). In Fig. 4A, nerve cells loss and obvious vacuolar changes were observed in anterior horn of injured spinal cord in the decompression group contrasted to the sham group. After gabapentin intervention for 4 weeks, the numbers of neurons were notably increased and vacuolar changes were suppressed by contrast to the vehicle control.

In order to observe axonal degeneration in each group, the axons in the anterior horn of spinal cord were measured by LFB staining (Fig. 4B). Contrasted to the sham group, obvious vacuolar changes and lower staining intensity were showed in the decompression group. Gabapentin administration for 4 weeks following decompression ameliorated vascular degeneration and enhanced intensity of tissue staining.

At the same time, the expression levels of caspase-3 in the anterior horn of spinal cord were measured by immunohistochemistry (Fig. 4C). Compared with the sham group, the levels of caspase-3 were significantly increased in the decompression group. There was no difference between the decompression group and the decompression + vehicle group. Through gabapentin administration, the expression levels of caspase-3 were notably inhibited when contrasted to the vehicle control.

### Gabapentin administration suppressed Iba-1 and α2δ2 expression in the damaged injured spinal cord following decompression

**Fig. 5:**
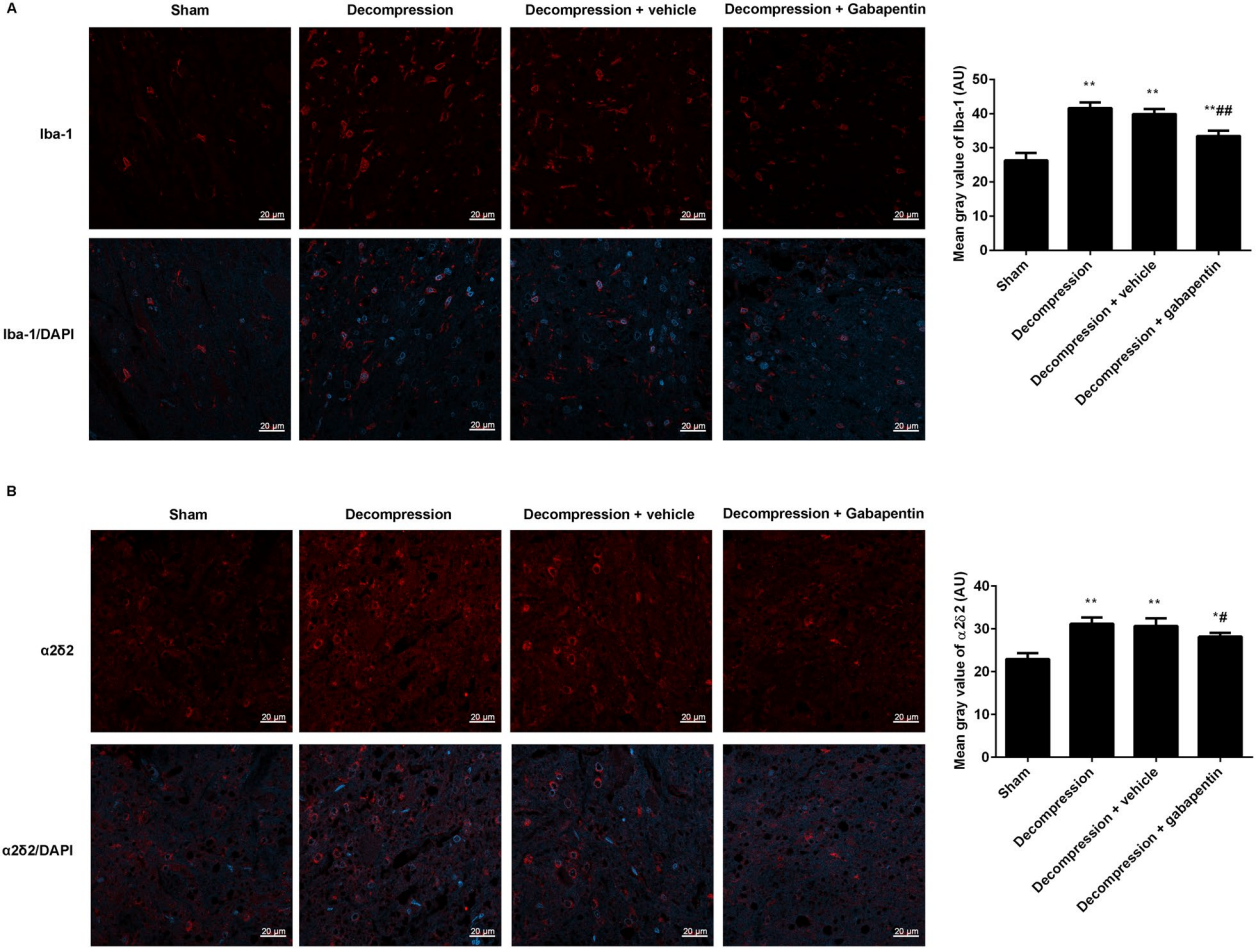
Gabapentin administration following decompression inhibited the expression levels of Iba-1 and α2δ2 in the anterior horn of spinal cord. **A** The expression of Iba-1 was observed by immunofluorescence (scale = 20 μm). **B** The expression of α2δ2 was observed by immunofluorescence (scale = 20 μm). The mean gray values of Iba-1 and α2δ2 were analyzed by Image J software. Compared with the sham group, ^*^P < 0.05, ^**^P < 0.01; compared with the decompression + vehicle group, ^#^P < 0.01, ^##^P < 0.01

In order to analyze the effects of gabapentin administration on Iba-1 and α2δ2 in the anterior horn of damaged spinal cord, their expressions were observed by immunofluorescence (Fig. 5). Contrasted to the sham group, the mean gray values of Iba-1 (Fig. 5A) and α2δ2 (Fig. 5B) were clearly increased in the decompression group. No difference was found between the decompression group and the decompression + vehicle group. Gabapentin intervention following decompression suppressed the expression of Iba-1 and α2δ2 when compared to the vehicle control.

### Gabapentin administration enhanced 5HT and GAP43 expression in the injured spinal cord following decompression

**Fig. 6:**
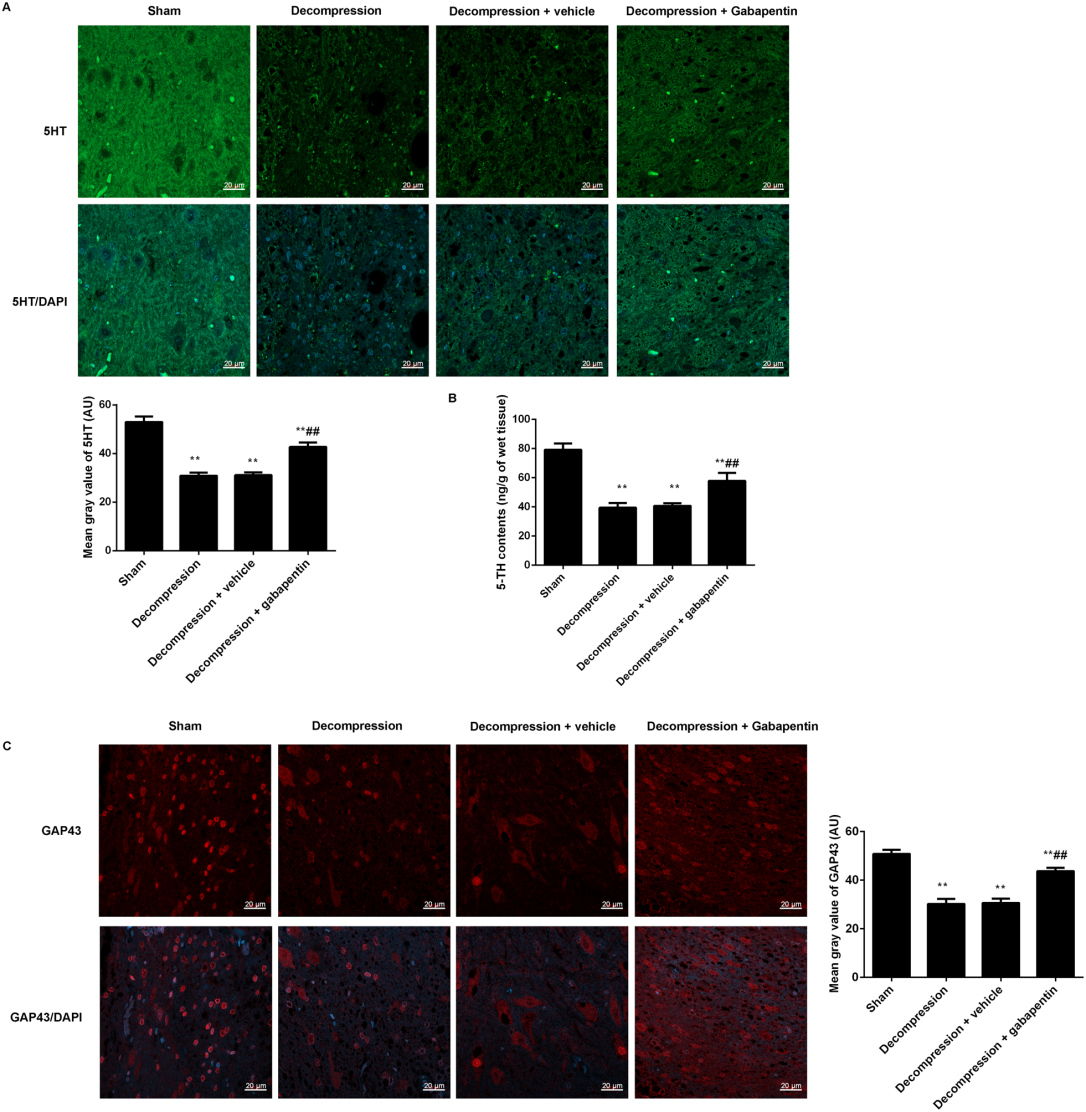
Gabapentin administration following decompression increased the expression of 5HT and GAP43 in the damaged spinal cord. **A** The expression of 5HT was observed by immunofluorescence (scale = 20 μm). **B** The levels of 5HT were observed by ELISA. **C** The expression of GAP43 was observed by immunofluorescence (scale = 20 μm). The mean gray values of 5HT and GAP43 were analyzed by Image J software. Compared with the sham group, ^**^P < 0.01; compared with the decompression + vehicle group, ^##^P < 0.01

Further to study the effects of gabapentin administration on 5HT and GAP43 expression in the damaged spinal cord following decompression, their levels were showed in Fig. 6. The expression levels of 5HT were analyzed by immunofluorescence (Fig. 6A) and ELISA (Fig. 6B), and the levels of 5HT were significantly declined in the decompression group compared with the sham group. Moreover, the levels of 5HT were obviously upregulated after gabapentin administration when contrasted to the vehicle control. The GAP43 expression levels were also observed by immunofluorescence (Fig. 6C), and its expression were similarly with the results of 5HT.

## Discussion

Recently, α2δ informs (α2δ2 and α2δ3) have been recognized as important regulators of synapse formation and plasticity [10, 17, 18]. Importantly, α2δ2 has showed a suppression of axonal regeneration in adult mice after spinal cord injury [10]. And, axonal plasticity underpins functional recovery of injured spinal cord following surgical decompression in a rat model of CSM [9]. α2δ2 is one target of widely prescribed antiepileptic and antiallodynic drugs, such as gabapentin and pregabalin. These important findings supported our findings that α2δ2 played a negative role in axonal recovery of CSM rats following surgical decompression, and α2δ2 pharmacological blockade through gabapentin treatment enhanced axonal plasticity following decompression.

5HT (serotonin) is known to mediate neurite growth responses [9, 19]. Under normal conditions, 5HT exposure leads to an approximately threefold increase in neurite growth rate, that depends on Ca-calcineurin-dependent cofilin activation and a PKC-dependent increase of myosin II activity in the growth cone rear [19]. GAP43 is known to play a key role in the regeneration and budding of axons, which is a marker of axon regeneration and new connection formation in nerve cells [20, 21]. It has reported that surgical decompression triggered a regenerative response in axons of CSM rats, and the expressions of 5HT and GAP43 in the spinal cord after decompression were higher than in the compression status [9]. In this study, we firstly showed that surgical decompression decreased the α2δ2 expression in the spinal cord of CSM rats, and α2δ2 might regulate 5HT and GAP43 expression in the axonal recovery of CSM following surgical decompression.

Ultimately, inhibition of α2δ2 expression through gabapentin treatment following surgical decompression improved the behaviors of rats when contrasted to the vehicle control in the CSM rats. Furthermore, gabapentin treatment suppressed the caspase-3 expression, Iba-1 activation and ameliorated axonal regeneration in the damaged spinal cord. The caspase family proteins, including caspase-3, caspase-7 and caspase-9 are closely related to nerve cells apoptosis, and involve in executing apoptosis [22, 23]. And, Iba-1 activation is closely linked with the caspase family proteins in spinal cords [24, 25], which is a critical mediator of inflammation. Our data suggested that gabapentin treatment decreased nerve cells apoptosis and inflammation following decompression in a rat model of CSM. Moreover, gabapentin intervention enhanced the expression of 5HT and GAP43 in the damaged spinal cord of CSM rats. Although originally designed as a γ-aminobutyric acid (GABA) analogue, gabapentin does not interact with either GABA receptors, rather it binds to the α2δ subunit of calcium channels [26]. Suzuki et al. found that the efficacy of gabapentin was determined by serotonergic excitatory pathway in spinal injury rats, and the powerful actions of gabapentin after neuropathy were blocked by 5HT(3) receptor antagonism [26]. Here, a closely association between gabapentin and 5HT was found following decompression, suggesting that targeting α2δ2 might be a novel treatment strategy to improve axonal regeneration of CSM following surgical decompression.

However, more studies are needed to assess other mechanisms of neural plasticity to understand the regeneration and functional recovery of CSM after surgical decompression. For example, it is still unclear whether the dorsal root ganglia microenvironment will affect the different regeneration capacities in the CSM following surgical decompression.

## Conclusions

In summary, α2δ2 limited axon regeneration in a rat model of CSM following surgical decompression. Targeting α2δ2 may be a novel treatment strategy to improve axonal regeneration of CSM following surgical decompression.

## Methods

### Experimental animals

In this experiment, adult male Sprague-Dawley rats (Jinan Pengyue Experimental Animal Breeding Co., Ltd, Jinan, China), weighting 300–400 g, were used. All animals were housed under a 12 h light/dark cycle with controlled temperature and humidity conditions, and allowed to free access to water and food.

### Chronic cervical cord compression injury and surgical decompression

Referring to reported descriptions [27, 28], rats were anaesthetized with intraperitoneal injection of pentobarbital (40 mg/kg), and a midline incision was made at the cervical area (C2-T2). After retracting the skin and superficial muscles, the rats underwent a C5-C6 laminectomy, and a chronic progressive compression device was inserted into the C5-C6. The compression device is a plexiglass flat plate (10 × 6 mm), with a 2 mm diameter screw hole in the center, a 1 mm diameter round hole at each corner (JiTian Bio, Beijing, China). A thread was threaded the interspinous ligament and sacral spinal muscle tendon, tied and fixed them, and then threaded the thread through the small holes around the plate to tie and fix the compression device. The screw (pitch of 0.4 mm, length of 10 mm and diameter of 2 mm) was precisely advanced 0.2 mm (one half turn) using a microscope. Then, the surgical wounds were sutured. Strict aseptic operation was performed during the operation, and penicillin (80 U/g) was injected to prevent infection for 5 days after the operation. The animals were given 0.9% saline (5 mL) to prevent dehydration, and housed in standard rat cages with 26 °C. After 1 week, the screw was advanced 0.2 mm every week, total 5 weeks. In contrast, the sham operated animals underwent identical surgical procedure but without having cord compression.

For surgical decompression, the same anesthesia methods were applied and the compression device was removed carefully. After surgery, the paravertebral muscle was sutured in layers, and the skin incision was stapled. Animals were received 0.9% saline and penicillin after surgery to prevent dehydration and infection, and housed in standard rat cages with 26 °C.

### Experimental groups

Design 1:

Thirty-two rats were used to evaluate the changes of α2δ2 expression in spinal cords from C5-C7 at compression 5 week (n = 6), decompression 7 d (n = 6), 14 d (n = 6), 21 d (n = 6) and 28 d (n = 6). One rat died on the next day after compression, and one rat died during the compression surgery.

**Fig. 7:**
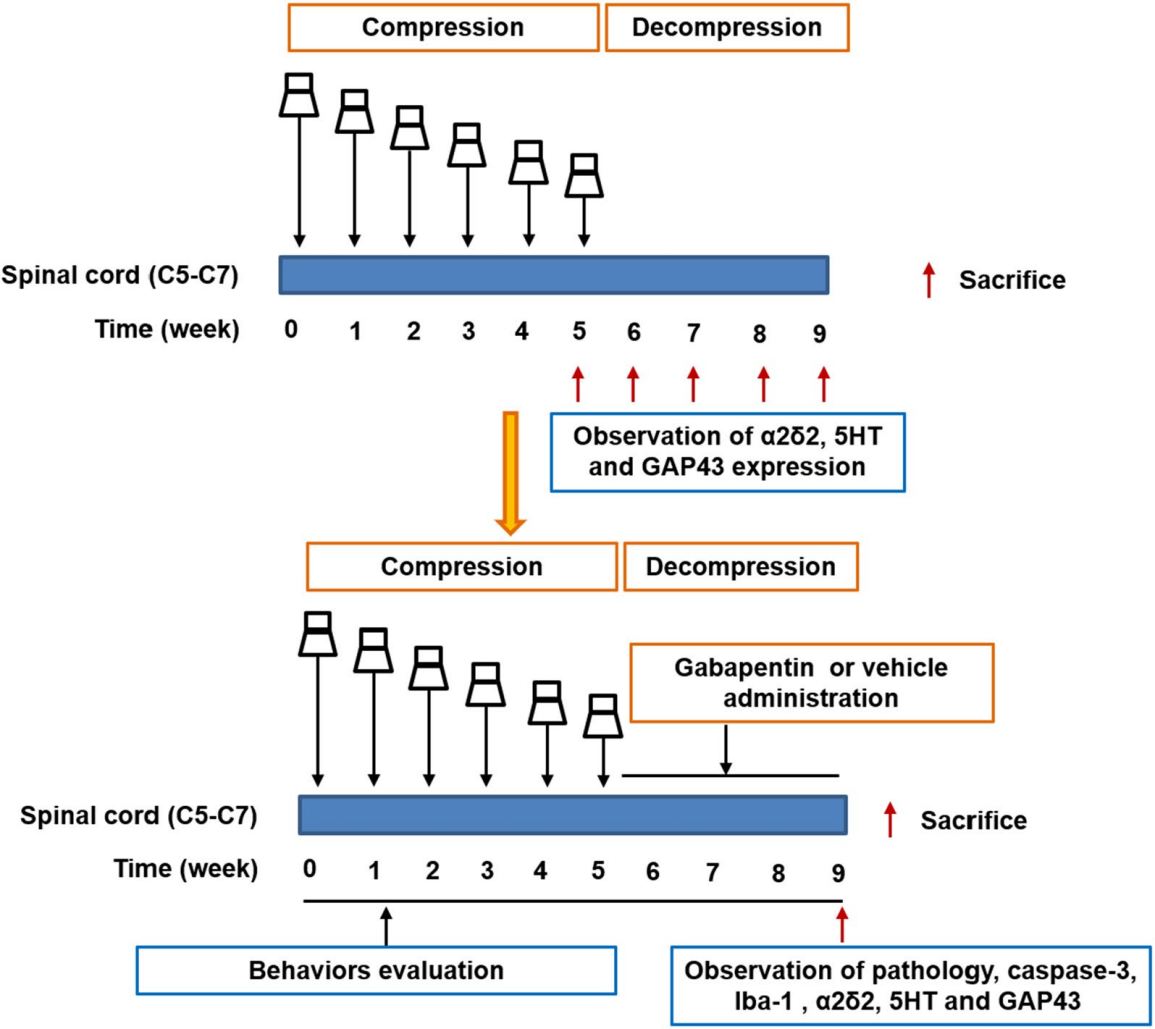
The experimental diagram in this study

Design 2:

Thirty-eight rats were randomly divided into the sham group (n = 9), CSM with decompression (Decompression, n = 9) group, CSM with decompression and vehicle (Decompression + vehicle, n = 9) group, CSM with decompression and gabapentin (Decompression + gabapentin, n = 9) group. One rat died during the compression surgery in the decompression + vehicle group and decompression + gabapentin group, respectively. The experimental diagram showed in the Fig. 7.

### Drug administration

After one hour of surgical decompression, gabapentin (46 mg/kg) was injected intravenously via tail vein, once a day until the end of the study [29]. In the decompression + vehicle group, the corresponding volume of 0.9% saline was injected as vehicle control. After 4 weeks decompression, this study was ended.

### Behaviors test

Basso, Beattie and Bresnahan (BBB) locomotor rating scale.

According to the 21-point BBB locomotor rating scale, joint movements, intervals of uncoordinated stepping, forelimb and hindlimb coordination were assessed in the process of CSM and surgical decompression. The evaluation was performed once a week by two examiners independently.

Inclined plane test.

Rats were placed on a smooth board. The board initially placed in a horizontal direction (0°), and the angle was increased by 5–10° after each attempt. The highest angle the rat could maintain for 8 s was recorded. The test was performed once a week.

### Samples collection

After behaviors evaluation, rats were sacrificed by intraperitoneal anesthesia with pentobarbital (40 mg/kg), and perfused with 4% paraformaldehyde. Fresh spinal cords from C5-C7 were collected, some fixed with 4% paraformaldehyde for overnight, and embedded with paraffin. Others were stored at -80 °C.

### Western blot

The tissues were washed with phosphate-buffered saline (PBS) and ground with lysate (g: mL = 1: 10) for 30 min. After centrifugation (10,000 ×*g*, 10 min), the concentration of protein in supernatant was measured using bicinchoninic acid assay (BCA) method. The proteins (50 µg, 10 µL) were added into 5 × SDS-PAGE solution to electrophoresis. When bromophenol blue ran to the gel boundary, the constant voltage was changed to 120 V from 80 V. The PVDF membrane was activated with methanol for 15 s, and infused it in water for 2 min. After immersing the PVDF membrane in membrane transfer buffer for 5 min, the proteins were transferred to the PVDF membrane. After transferring, the membrane was washed 3 times with TBS-T and blocked with 5% skimmed milk for 90 min. According to the instructions, the primary antibodies including α2δ2 (#PA5-77341, 1:200, ThermoFisher Science, China) and GAPDH (#PA1-988, 1:800, ThermoFisher Science, China), were diluted using TBS-T with 5% skimmed milk, and incubated at 4 °C for overnight. After washing the membrane with TBS-T for 3 times, the Goat anti-Rabbit lgG (H + L) second antibody (#31,460, 1:200, ThermoFisher Science, China) diluted by TBS-T with 5% skimmed milk, was added to culture for 90 min at room temperature. After washing, the membrane was colored with enhanced chemiluminescence (ECL) reagent. The quantitative analysis of proteins was analyzed by ImageJ software (National Institutes of Health).

### Hematoxylin-eosin (HE) staining

The embedded tissues were cut into 3 μm, then dewaxed with xylene (2 times, 5 min each time), and hydrated with graded ethanol (ethanol, 5 min; 95% ethanol, 2 min; 80% ethanol, 2 min; 70% ethanol, 2 min). After washing with water for 2 min, the sections were stained with hematoxylin staining (G1120, Solarbio, Beijing, China) for 15 min, then differentiated with differentiation liquid for 30 s. After soaking for 15 min in water, the sections were stained with eosin (G1120, Solarbio, Beijing, China) for 50 s. After washing and soaking with water, the sections were dehydrated with graded ethanol (95% ethanol, 2 s; 95% ethanol, 2 s; 100% ethanol, 2 s; 100% ethanol, 1 min;) and purified with xylene (2 times, 1 min each time). At least, the sections were sealed with neutral gum and observed under a light microscope (DM1000 LED, Leica, Germany).

### Immunofluorescence

The embedded tissues were baked for 20 min at 60 °C, then dewaxed and hydrated as described in HE staining. The sections were placed in 0.01 mol/L sodium citrate buffer (pH = 6.0) and heat them boiling using microwave oven (repeating 2 times at an interval of 10 min). After washing with 0.01 mol/L PBS (pH = 6.0) for 3 times, 5 min each time, the sections were cultured with 3% H_2_O_2_ for 10 min, then washed with PBS for 3 times. The primary antibodies including α2δ2 (#PA5-77341, 1:400, ThermoFisher Science, China), 5 hydroxytryptamine (5HT, #ab6336, 1:1000, Abcam, China), GAP43 (#ab277627, 1:1000, Abcam, China), Iba-1 (#ab178847, 1:100, Abcam, China) were added to the sections and cultured for overnight at 4 °C. After washing with PBS for 3 times, the goat secondary antibody goat (1:500, ThermoFisher Science, China) were added to culture for 60 min at 37 °C. Then, the sections were counterstained with 4’,6-diamidino-2-phenylindole (DAPI) for 5 min. After washing with PBS and quenching fluorescence, the sections were observed under a laser confocal microscope (LSM800, Zeiss, Germany).

### ELISA for 5HT

The spinal cord tissues were homogenized in 0.1 M PBS containing 0.1% ascorbic acid, and then centrifuged (10,000 ×*g*) for 20 min at 4 °C to collect supernatant. The levels of 5HT were analyzed by an ELISA kit (E-EL-003c, Elabscience, China). 5-HT levels were normalized to wet tissue weight.

### Luxol fast Blue (LFB) staining

The embedded sections were dewaxed and hydrated as described in HE staining, and stained with LFB staining (G3245, Solarbio, China) following kit instructions. After washing, the sections were differentiated to colorless in the background. The sections were dehydrated with ethanol and cleared with xylene. Neutral gum was used to seal the pieces. The results were observed under a light microscope.

### Immunohistochemistry

Similar experimental steps with the immunofluorescence, the primary antibody caspase-3 (#PA5-77887, 1:800, ThermoFisher Science, China) was added to culture for overnight at 4 °C. Then the sections were cultured with the Goat anti-Rabbit lgG (H + L) second antibody (#31,460, 1:1000, ThermoFisher Science, China) for 60 min at 37 °C. The sections were stained with the diaminobezidin (DAB) solution at the room temperature. After washing with water, the sections were counterstained with hematoxylin for 60 s. After the dehydration, purification and seal, the results were observed under a light microscope.

### Statistical analysis

All data was processed by SPSS 20.0 statistical analysis software (IBM, Chicago, IL, USA) and the results were expressed as the mean ± standard deviation. One-way analysis of variance was exerted for data analysis among groups, followed by LSD test. P < 0.05 means a significant.

## Data Availability

All data generated in this published article and its supplementary information files for further data it can be available with corresponding author.
